# SNP-based selection strategies reveal antagonistic and favorable genetic architectures between milk production and fertility traits in dairy cattle: A simulation study

**DOI:** 10.1016/j.vas.2026.100748

**Published:** 2026-06-23

**Authors:** Heydar Ghiasi, Navid Ghavi Hossein-Zadeh

**Affiliations:** a Department of Animal Science, Faculty of Agricultural Science, Payame Noor University, Tehran, 19395-4697, Iran; b Department of Animal Science, Faculty of Agricultural Sciences, University of Guilan, Rasht, Iran

**Keywords:** Genomic selection, SNP effects, Milk production, Fertility, Genetic gain, GWAS, Pleiotropy, Selection response, Multi-trait selection

## Abstract

This study evaluated genetic responses to selection based on single-nucleotide polymorphism (SNP) subsets with different effect directions on milk production and fertility in dairy cattle. A population of 1000 individuals was simulated using 50,000 SNP markers sampled from the Illumina BovineSNP50 BeadChip panel to represent a realistic genomic architecture. SNP effects were derived from previously published genome-wide association study (GWAS) results, in which the direction of SNP effects was inferred from reported test statistics (t-values). Five selection scenarios were evaluated over 50 generations, including selection based on milk-only SNPs, fertility-only SNPs, antagonistic SNP combinations, and SNPs with favorable effects on both traits. Results showed that single-trait selection maximized genetic gain for the target trait, with the milk_only and fert_only scenarios achieving the highest responses for milk production and fertility, respectively. Antagonistic SNP scenarios generated asymmetric trade-offs, where improvement in one trait was accompanied by deterioration, or where selection failed to improve the intended trait despite modest progress in the correlated trait. In contrast, selection based on SNPs with favorable effects on both traits produced simultaneous and sustained improvement in milk production and fertility, although at lower rates than single-trait selection. Genetic correlations were positive under favorable scenarios but strongly negative under antagonistic scenarios, indicating persistent genetic conflict between traits. These findings demonstrate that SNP effect direction and pleiotropy play a critical role in shaping long-term genetic responses and highlight the importance of multi-trait genomic selection strategies for achieving balanced and sustainable genetic improvement.

## Highlights

• A simulation framework used GWAS-based SNP effect directions.
• Antagonistic SNPs caused trade-offs and negative genetic correlations.
• Single-trait selection maximized gain but harmed correlated traits.
• Favorable SNPs enabled balanced improvement in milk and fertility.
• Highlights role of SNP direction and pleiotropy in genomic selection.

## Introduction

Genetic improvement programs in dairy cattle have historically focused on increasing milk production because of its major economic importance. However, long-term selection for higher milk yield has often been accompanied by reduced fertility and reproductive performance, resulting in an unfavorable genetic relationship between these traits (Berry et al., 2014; Pryce et al., 2002).

The development of genomic selection has revolutionized animal breeding by enabling the use of dense single-nucleotide polymorphism (SNP) markers across the genome to predict genetic merit. Early studies demonstrated that genome-wide marker information can substantially improve the accuracy of estimated breeding values (Meuwissen et al., 2001; VanRaden, 2008; VanRaden et al., 2009). As a result, genomic selection has become a fundamental component of modern dairy cattle breeding programs.

Genome-wide association studies (GWAS) have identified numerous loci associated with milk production, fertility, and other economically important traits (Cole et al., 2011). Many of these loci exhibit pleiotropic effects, such that some SNPs have favorable effects on both traits, whereas others improve one trait while adversely affecting the other. Consequently, the direction of SNP effects may play a critical role in determining the efficiency and sustainability of genomic selection programs. Although the unfavorable genetic relationship between milk production and fertility has been extensively documented, limited research has investigated how different classes of SNPs, defined according to the direction of their effects across traits, influence long-term genetic responses under genomic selection. In particular, no previous study has systematically compared favorable pleiotropic SNPs, antagonistic SNPs, and single-trait-associated SNPs using a GWAS-based simulation approach. Therefore, the objective of this study was to evaluate long-term genetic gain, genetic correlations, and trade-offs between milk production and fertility under alternative SNP-based selection scenarios. By comparing genomic selection strategies based on SNP subsets with different effect directions, this study provides an exploratory framework to investigate how pleiotropy and SNP-effect direction may influence long-term genetic responses and the sustainability of multi-trait breeding programs.

## Materials and methods

### SNP Data source and marker panel

A panel of single-nucleotide polymorphisms (SNPs) was constructed based on the Illumina BovineSNP50 BeadChip, which represents a widely used medium-density genotyping platform in dairy cattle. The SNP list, including marker identifiers, chromosomal positions, and allelic information, was downloaded from the EnsemblBiomart (Kinsella et al., 2011) and preprocessed to retain autosomal markers. From this panel, a total of 50,000 SNPs were used in the simulation. The selection of markers followed a two-step approach. First, SNPs identified as significantly associated with milk production and fertility traits (daughter pregnancy rate considered as a fertility trait) were extracted from previously published genome-wide association study (GWAS) results (Cole et al., 2011). These SNPs were ensured to be included in the simulated panel. Second, the remaining SNPs were randomly sampled from the BovineSNP50 panel to reach a total of 50,000 loci. This strategy ensured that the simulated genome maintained both biological relevance and sufficient background variation.

### Definition of traits and GWAS-derived SNP effects

Two traits were considered in this study: milk production (MY) and fertility (FERT). The milk production trait (MY) represents milk yield, while the fertility trait (FERT) was constructed as a composite genetic trait based on SNP effects associated with daughter pregnancy rate (DPR) obtained from genome-wide association study (GWAS) results. It is important to note that neither MY nor FERT represents directly simulated phenotypic measurements; instead, both traits reflect the underlying genetic architecture inferred from SNP associations. GWAS summary statistics were used to define SNP effects for both traits. Specifically, t-values associated with each SNP were used to determine the direction of allelic effects, where positive t-values indicate favorable effects and negative t-values indicate unfavorable effects. Because direct effect sizes (β coefficients) were not available, SNP effects were sampled from a standard normal distribution N (0,1). The sign of each simulated effect was determined according to the sign of the corresponding GWAS t-value. Positive t-values generated positive SNP effects, whereas negative t-values generated negative SNP effects. A normal distribution was selected as a simple and neutral assumption that assigns equal prior probability to positive and negative effect magnitudes and avoids imposing a specific genetic architecture. Although alternative distributions, such as Gamma distributions or Bayesian mixture priors used in BayesA and BayesB, may better represent the heavy-tailed distribution of SNP effects observed in some complex traits, the objective of the present study was not to model the exact distribution of SNP effect sizes but rather to investigate the consequences of SNP-effect direction and pleiotropy on long-term selection responses. Separate effect vectors were generated for MY and FERT. The GWAS results used in this study were derived from previously published analyses of dairy cattle traits, including milk production and fertility-related traits such as DPR (Cole et al., 2011). Therefore, SNP inclusion was based on the significance criteria reported in the original study. The present study did not apply an additional statistical threshold; instead, SNPs were classified according to the sign of the reported GWAS t-values for MY and FERT. Therefore, the simulated traits in this study represent genetic constructs derived from GWAS-based association signals rather than observed phenotypic records.

### Genotype simulation

A population of 1000 individuals was simulated. Minor allele frequencies (MAF) for SNPs were sampled from a uniform distribution between 0.2 and 0.5. Genotypes were generated assuming Hardy–Weinberg equilibrium using binomial sampling, resulting in genotype values coded as 0, 1, or 2. All SNPs were assumed to be independent (linkage disequilibrium was not modeled).

### Selection scenarios

Five SNP-based selection scenarios were defined according to the direction of SNP effects on milk production (MY) and fertility. In the first scenario (milk_only), selection was based exclusively on SNPs associated with MY. In the second scenario (fert_only), selection was based on SNPs associated with fertility. The third scenario (milk_pos_fert_neg) included SNPs with favorable effects on MY and unfavorable effects on fertility, representing an antagonistic genetic structure. Conversely, the fourth scenario (fert_pos_milk_neg) consisted of SNPs with favorable effects on fertility and unfavorable effects on MY. Finally, the fifth scenario (both_positive) included SNPs with favorable effects on both MY and fertility, representing a favorable pleiotropic genetic architecture. These scenarios enabled the evaluation of genetic responses under both synergistic and antagonistic relationships between milk production and fertility traits. The antagonistic and favorable pleiotropic SNP subsets (milk_pos_fert_neg, fert_pos_milk_neg, and both_positive) were mutually exclusive and were defined according to the sign of SNP effects on both traits. In contrast, the milk_only and fert_only scenarios included all GWAS-derived SNPs and differed only in the selection criterion used for ranking individuals. Thus, pleiotropic SNPs were retained in these scenarios and contributed to breeding value calculation whenever they were present among the GWAS-derived loci.

The distribution of GWAS-derived SNPs across the five selection scenarios is presented in Table 2. A total of 1427 SNPs were identified from GWAS results and classified according to the direction of their effects on milk production and fertility. Among these, 460 SNPs showed favorable effects on both traits (both_positive), whereas 284 and 294 SNPs exhibited antagonistic effects in the milk_pos_fert_neg and fert_pos_milk_neg scenarios, respectively.

### Breeding value calculation, selection and mating, and simulation design

Simulated total breeding values (TBV) for milk production (MY) and fertility were derived from SNP genotypes and their corresponding SNP effects across all loci included in each scenario according to:

$$TBV_{i,t}=\;\sum_{j=1}^{m}G_{ij}\beta_{jt}$$

$TBV_{i,t}$ = Total breeding value of individual i for trait t, $G_{ij}$ = Genotype of individual i for SNP j (coded as 0, 1, or 2), and $\beta_{jt}$ = SNP effect of SNP j on trait t.

To prevent one trait from numerically dominating the selection index, raw TBVs were standardized to account for differences in trait variance and heritability (h² = 0.30 for MY and h² = 0.05 for fertility), while raw TBVs were retained for evaluating genetic gain. Specifically, TBVs were centered and scaled by their standard deviation and subsequently weighted by the square root of trait heritability. The standardized breeding values were then combined to construct the multi-trait selection index. This procedure ensured that differences in scale between MY and FERT did not disproportionately influence selection decisions.

$$TBV_{stan}=\left(\frac{TBV-\bar{TBV}}{\sigma_{TBV}}\right)\times \;\sqrt{h^{2}}$$

$TBV_{stan}$ = standardized total breeding value, TBV = total breeding value, $\bar{TBV}$ = mean TBV of the population in the corresponding generation,$\;\sigma_{TBV}$= standard deviation of TBV within the population, $h^{2}\;=\;$heritability of the trait.

Heritability was used only to scale standardized breeding values for selection purposes. Environmental and residual effects were not simulated; therefore, heritability did not influence phenotype generation and was used solely to balance the contribution of traits in the selection index. In each generation, individuals were ranked based on scenario-specific selection criteria, and the top 20% were selected as parents. Random mating was then implemented among selected individuals to generate the next generation, with offspring genotypes simulated based on parental allele contributions. Sex was not explicitly modeled in the simulation. Selected parents were sampled randomly from the selected population, and mating pairs were generated without assigning individuals to male or female categories. Discrete generations were assumed. The simulation was conducted over 50 generations, and in each generation, mean TBVs for MY and fertility, genetic gain per generation (ΔG), cumulative genetic gain, and genetic correlations between traits were calculated. Correlations between milk and fertility breeding values were estimated using Pearson correlation coefficients among individual simulated TBVs and were used as indicators of the association between breeding values across traits. This framework allowed evaluation of both direct and correlated responses to selection under different SNP-based scenarios. All simulations were implemented in Python 3.8 using the NumPy and Pandas libraries. All simulation codes are provided in Supplementary file 1.

### Interpretation of genetic values

It should be noted that the total breeding values (TBVs) and genetic gain (ΔG) reported in this study were derived from simulated SNP effects and do not correspond directly to real phenotypic measurements. The simulated values represent relative genetic responses to selection and are expressed in arbitrary units. Therefore, the magnitude of TBV and ΔG should be interpreted in terms of direction and comparative response across selection scenarios rather than as absolute trait values.

## Results

### Genetic gain for milk production

**Fig. 1:**
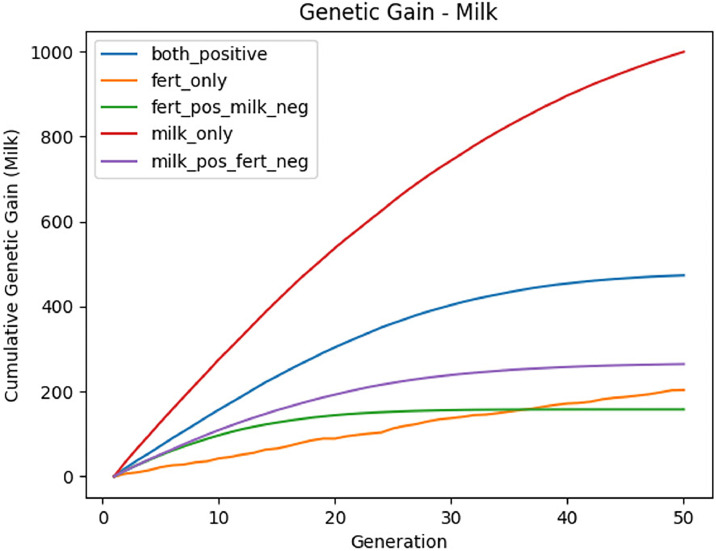
Cumulative genetic gain for milk production across 50 generations under five SNP-based selection scenarios.

Genetic gain for milk production increased across generations in all scenarios; however, the magnitude of response differed markedly among selection strategies. These patterns are illustrated in Fig. 1. In the milk_only scenario, mean TBV increased from 24.84 in generation 1 to 1023.83 in generation 50, corresponding to the highest response with an average ΔG of 20.39 units per generation. In the both_positive scenario, milk TBV increased from 256.02 to 729.17, with an average ΔG of 9.66, indicating a stable and balanced response across generations. The milk_pos_fert_neg scenario showed a moderate increase in milk TBV (147.05 to 411.50), with ΔG of 5.39, reflecting reduced efficiency due to antagonistic SNP effects. The fert_only scenario resulted in a limited increase in milk TBV (24.84 to 228.19), with ΔG of 4.15. The fert_pos_milk_neg scenario showed weak recovery in milk TBV (−157.61 to ∼0), with ΔG of 3.22 (Table 1).

**Table 1:** Genetic gain, average response per generation (ΔG), and **correlation between simulated TBVs**f or milk production and fertility across selection scenarios.

Scenario	Milk (Gen1)	Milk (Gen50)	Δ G Milk (avg/gen)	Fert (Genl)	Fert (Gen50)	Δ G Fert (avg/gen)	Genetic correlatio n Gen1	Genetic correlatio n Gen50
milk_only	24.84	1023.8 3	20.39	49.39	184.41	2.76	0.19	0.08
fert_only	24.84	228.19	4.15	49.39	1064.0 2	20.72	0.19	0.05
both_positive	256.0 2	729.17	9.66	243.6 1	688.88	9.09	0.65	0.34
milk_pos_fert_ne g	147.0 5	411.50	5.39	153.4 3	−412.12	−5.28	−0.60	−0.93
fert_pos_milk_ne g	157.6 1	0.00	3.22	173.5 6	2.04	−3.50	−0.55	−1

Milk (Gen1) and Milk (Gen50), and Fert (Gen1) and Fert (Gen50), represent the mean true breeding values for milk production and fertility at generations 1 and 50, respectively. ΔG (avg/gen) denotes the average genetic gain per generation. Correlation among simulated TBVs is reported for generations 1 and 50.

**Table 2:** Distribution of GWAS-derived SNPs across selection scenarios according to the direction of their effects on milk production (MY) and fertility (FERT).

Scenario	MY effect	FERT effect	Number of SNPs
milk_only	All MY-associated SNPs	Mixed	1427
fert_only	Mixed	All FERT-associated SNPs	1427
milk_pos_fert_neg	Positive	Negative	284
fert_pos_milk_neg	Negative	Positive	294
both_positive	Positive	Positive	460

MY = milk production trait; FERT = fertility trait. SNPs were classified according to the sign of GWAS-derived t-values obtained from published GWAS results. Positive and negative effect directions indicate favorable and unfavorable associations with the corresponding trait, respectively. Mixed indicates that SNPs were not classified according to the direction of effects on that trait and could include loci with either positive or negative effects. The Number of SNPs column represents the number of GWAS-derived loci included in each selection scenario after classification according to their effects on both milk production and fertility. The milk_only and fert_only scenarios included all GWAS-derived SNPs, whereas the remaining scenarios consisted of SNP subsets with specific combinations of effect directions across traits.

### Genetic gain for fertility

**Fig. 2:**
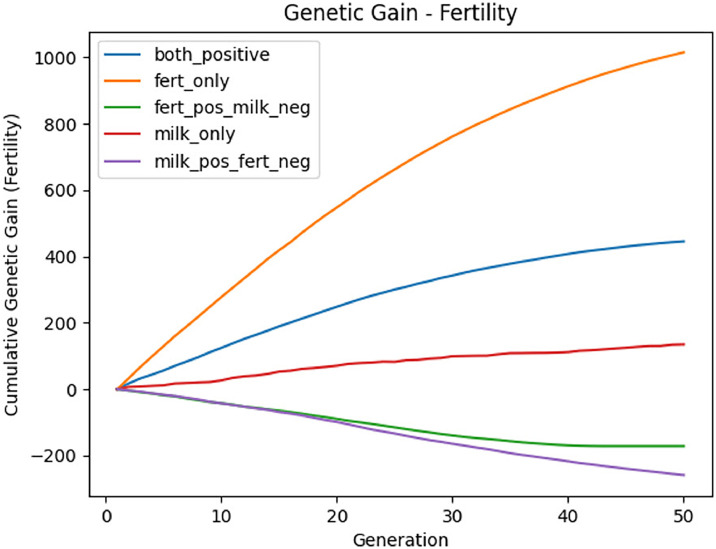
Cumulative genetic gain for fertility across 50 generations under different selection strategies.

Fertility responses showed a contrasting pattern. These trends are shown in Fig. 2. The fert_only scenario achieved the greatest improvement, with TBV increasing from 49.39 to 1064.02 and ΔG of 20.72. The both_positive scenario also showed strong improvement (243.61 to 688.88), with ΔG of 9.09. In contrast, the milk_pos_fert_neg scenario showed a strong decline in fertility (−153.43 to −412.12), with ΔG of −5.28. The milk_only scenario resulted in modest improvement (49.39 to 184.41), with ΔG of 2.76. The fert_pos_milk_neg scenario showed a decline in fertility (173.56 to 2.04), with ΔG of −3.50 (Table 1).

### Correlation trends between milk production and fertility across selection scenarios

**Fig. 3:**
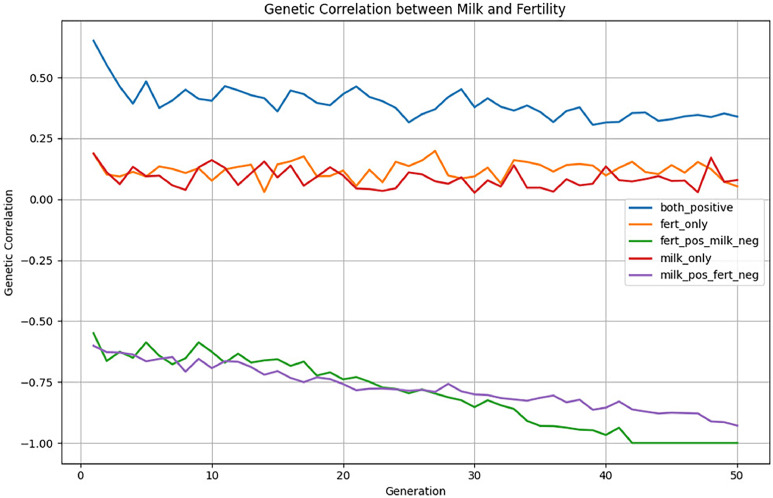
Genetic correlation trends between milk production and fertility across generations.

Correlations between simulated breeding values for milk production and fertility varied markedly among selection scenarios across generations (Fig. 3). In the both_positive scenario, correlations remained positive throughout, decreasing slightly from approximately 0.65 in generation 1 to 0.34 in generation 50, reflecting initially aligned SNP effects and a gradual reduction in synergy between traits. Although all loci in this scenario had favorable effects on both traits, differences in effect magnitudes between milk production and fertility resulted in unequal responses to allele-frequency changes across generations. Consequently, the covariance between traits gradually declined, leading to a moderate reduction in breeding-value correlations while maintaining an overall favorable relationship. In the milk_only scenario, correlations remained low but positive, ranging from 0.19 in generation 1 to 0.08 in generation 50. Similarly, the fert_only scenario showed modest positive correlations (0.19 to 0.05). In contrast, the milk_pos_fert_neg scenario exhibited strong negative correlations from −0.60 in generation 1 to −0.93 in generation 50, highlighting pronounced antagonistic effects between milk and fertility. For the fert_pos_milk_neg scenario, correlations started at −0.55 in generation 1 and gradually approached −1, reaching −1 from generation 42 onwards, indicating a strong and sustained negative relationship (Table 1). Overall, these patterns demonstrate that the direction of SNP effects critically determines whether selection produces synergistic or antagonistic genetic responses between milk production and fertility.

### Trade-off between milk production and fertility

**Fig. 4:**
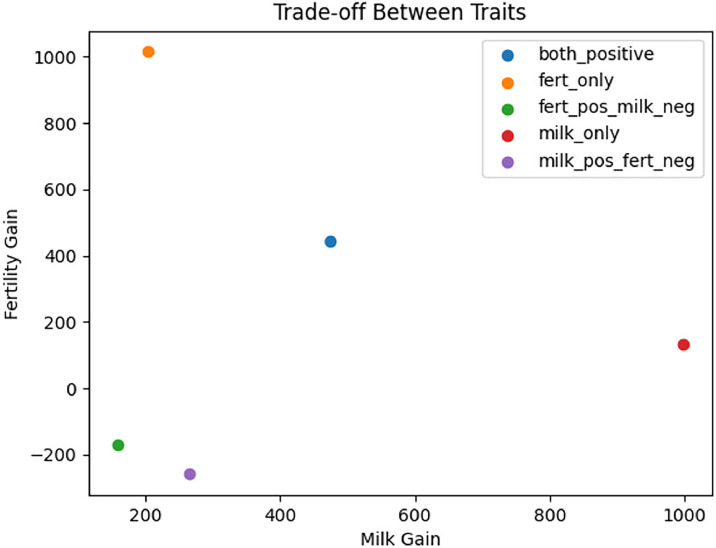
Trade-off between milk production and fertility at generation 50 across scenarios.

The trade-off between milk production and fertility was clearly illustrated by the distribution of scenarios in two-trait response space (Fig. 4). The both_positive scenario occupied the most favorable region, achieving simultaneous positive gain for both traits. In contrast, milk_only and fert_only maximized gain in one trait but showed limited response in the other. Antagonistic scenarios (milk_pos_fert_neg and fert_pos_milk_neg) were located in regions with negative response for one trait, confirming strong genetic trade-offs.

### Overall comparison

Single-trait selection scenarios maximized genetic gain for their respective target traits, whereas the both_positive scenario produced more balanced responses across milk production and fertility. In contrast, antagonistic scenarios resulted in asymmetric responses, reflecting trade-offs between traits rather than simultaneous improvement.

## Discussion

The present study demonstrated that SNP-based selection strategies can generate markedly different patterns of genetic gain depending on the direction and combination of marker effects across traits. The superiority of the milk_only and fert_only scenarios for their respective target traits is consistent with the principles of directional selection, whereby maximizing selection pressure on a single trait yields the greatest genetic response (Falconer & Mackay, 1996). However, such strategies resulted in limited or unfavorable correlated responses in the alternative trait, highlighting the biological constraints of single-trait improvement.

Antagonistic genetic relationships between milk production and fertility have been widely reported in dairy cattle. Long-term selection for higher milk yield has frequently been associated with poorer reproductive performance, including lower conception rate and longer calving interval (Lucy, 2001; Pryce et al., 2004). Similar evidence has been reported in Holstein cattle, where production-oriented selection generated unfavorable correlated responses in fertility traits (Ghiasi et al., 2013). Although genomic selection based on bovine SNP chips offers new opportunities for simultaneous improvement of multiple traits, such antagonism may persist because some loci have favorable effects on milk production but unfavorable effects on fertility, whereas others show the opposite pattern (Cole et al., 2011). This mechanism was clearly reflected in the antagonistic scenarios of the present study. In milk_pos_fert_neg, genetic correlations became progressively more negative, changing from −0.60 to −0.93, whereas in fert_pos_milk_neg, they intensified from −0.55 to −1.00 from generation 42 onward, indicating increasingly strong genetic antagonism between milk production and fertility. An unexpected pattern was observed in the fert_pos_milk_neg scenario, where fertility declined despite the inclusion of SNPs with positive effects on fertility. This result can be explained by the multi-trait selection index used in the simulation, which combined standardized breeding values for both milk production and fertility. Because all SNPs in this scenario simultaneously carried unfavorable effects on milk production, the antagonistic genetic architecture limited the persistence of favorable fertility responses across generations and ultimately reduced fertility gain. In contrast, the both_positive scenario demonstrated that simultaneous improvement in milk production and fertility is achievable when selection is restricted to SNPs with favorable effects on both traits. Although responses were lower than those obtained under single-trait selection, gains in both traits were stable and balanced, supporting previous reports that inclusion of functional traits in breeding objectives improves long-term breeding efficiency (Berry et al., 2014).

It should be noted that the absolute TBV values reported here do not correspond directly to observed phenotypic measurements, as both traits were simulated using SNP-derived effects rather than recorded phenotypes. Therefore, the magnitude of response should be interpreted as relative genetic progress rather than direct phenotypic prediction. Overall, these results provide a theoretical illustration of how SNP effect direction may influence long-term responses to multi-trait genomic selection. Because the study was based on a simplified simulation framework, the findings should not be interpreted as direct evidence for practical breeding programs but rather as a basis for future investigations using real genomic and phenotypic data.

### Limitations of the study

Several limitations of the present study should be acknowledged. The simulation assumed independent SNPs and did not model linkage disequilibrium or environmental and residual effects, which simplifies the genetic architecture compared to real populations. In addition, SNP effects were generated based solely on the sign of GWAS-derived t-values rather than estimated effect sizes (β coefficients), and the classification of SNPs as favorable or unfavorable may depend on allele coding and trait definition.

Furthermore, total breeding values (TBV) and genetic gain (ΔG) were expressed in arbitrary units derived from simulated SNP effects and therefore do not correspond to observed phenotypic measurements. The simulation results were obtained from a single run without replication, which limits the assessment of variability across simulations.

In addition, the present study did not explicitly monitor changes in genetic variance, allele frequencies, fixation rates, or inbreeding across generations. Therefore, although long-term selection would be expected to reduce genetic diversity in a closed population, the magnitude of these effects could not be quantified in the current simulation. Future studies should investigate how alternative SNP-based selection strategies influence the maintenance of genetic variation and the rate of allele fixation over time.

Therefore, the results should be interpreted as theoretical outcomes under a simplified genomic framework rather than as direct predictions for practical breeding programs. Further studies using real genomic data, estimated SNP effects, and more realistic breeding scenarios are required to evaluate the practical implications of these findings.

### Strengths of the study

Strengths of the present study include the use of a realistic bovine SNP-chip framework, the incorporation of GWAS-derived SNP-effect directions, and the comparison of multiple SNP-based selection strategies under a common simulation environment. In addition, the evaluation of genetic responses over 50 generations allowed assessment of long-term selection consequences that are difficult to observe in real breeding programs. The study also provides a simple framework for investigating the effects of pleiotropy and SNP-effect direction on multi-trait genomic selection.

## Conclusion

SNP-based selection strategies produced markedly different patterns of simulated genetic response depending on the direction of SNP effects across milk production and fertility traits. Single-trait selection scenarios generated the highest response for their respective target traits, whereas antagonistic SNP subsets were associated with unfavorable correlated responses. In contrast, selection based on SNPs with favorable effects on both traits resulted in a more balanced improvement of milk production and fertility.

Because this study was based on a simplified simulation framework using GWAS-derived SNP-effect directions rather than estimated SNP effect sizes, the results should be interpreted as theoretical illustrations of potential long-term selection outcomes rather than direct predictions for practical breeding programs. Nevertheless, the findings suggest that SNP-effect direction may be an important factor to consider in multi-trait genomic selection and warrant further investigation using real genomic and phenotypic data.

## Funding information

This research received no funding.

## Ethics statement

Not applicable: This manuscript does not include human or animal research.

## CRediT authorship contribution statement

**Heydar Ghiasi:** Writing – review & editing, Writing – original draft, Validation, Supervision, Project administration, Investigation, Formal analysis, Conceptualization. **Navid Ghavi Hossein-Zadeh:** Writing – review & editing, Writing – original draft, Validation.

## Declaration of competing interest

The authors declare that they have no known competing financial interests or personal relationships that could have appeared to influence the work reported in this paper.

## Data Availability

The authors confirm that the data and simulation codes supporting the findings of this study are available within the article and its supplementary materials.

## Appendix Supplementary materials

**Supplementary file 1.** The simulation codes used in this study. Image, application 1
